# Microneedling combined with botulinum toxin-A versus microneedling combined with platelet-rich plasma in treatment of atrophic acne scars: a comparative split face study

**DOI:** 10.1007/s00403-022-02446-9

**Published:** 2022-11-05

**Authors:** Waleed Albalat, Soheir Ghonemy, Ayat Saleh, Mona Elradi

**Affiliations:** Dermatology, Venereology & Andrology Department, Zagazig University Hospitals, Zagazig University, Zagazig, Egypt

**Keywords:** Acne, Scars, Microneedling, PRP, Botulinum toxin-A

## Abstract

**Background:**

Atrophic post-acne scarring constitutes a troublesome cosmetic concern for both patients and dermatologists. Old and new therapies as well as combinations are being introduced to achieve a satisfactory response. Microneedling has been used either alone or under different combinations for its treatment. The aim was to compare its combination with topical platelet-rich plasma versus its combination with topical Botulinum Toxin-A.

**Methods:**

30 subjects with different types and grades of atrophic post-acne scars completed the study. Right side of the face was treated with microneedling and platelet-rich plasma while the left side was treated microneedling and Botulinum toxin-A. Response was assessed using two different scales. Patient satisfaction and pain were also assessed.

**Results:**

Regarding response to therapy and according to the quartile grading scale, there was no statistically significant difference between the two sides where (23.4% & 13.3%) of the right and left sides, respectively, had an excellent response. Regarding the difference in the qualitative global scarring grading system before and after treatment, there was a highly statistically significant improvement on both sides with higher improvement on the right side than left side but in a non-statistically significant way.

**Conclusions:**

Both combinations present efficacious options for treating acne scars with comparable efficacy.

**Trial registration:**

Registered and approved prospectively by the ethical review board of the faculty of medicine, Zagazig University.

## Introduction

Acne is a prevalent multifactorial skin problem that affects areas of the body that are rich in pilosebaceous glands, e.g., face, chest and upper back in the form of inflammatory and non-inflammatory lesions. Acne is known to be negatively impacting the patients’ quality of life being a troublesome cosmetic problem during its flares and because of the post-acne residuals, e.g., post-acne scars and dyspigmentation [1].

Post-acne scars are a permanent patient-distressing sequela of acne that doesn’t fade with time. It even becomes worse with skin aging. Acne scars usually result from the inflammatory acne lesions and their severity correlates with acne severity, duration, and the delay of management. Their exact prevalence and severity have not yet been established [2].

Acne scars are broadly classified into macular (erythematous and hyperpigmented), atrophic (ICE-pick, boxcar and rolling) and elevated (hypertrophic, keloidal, papular and bridging) scars. Different types usually coexist in the same patient making differentiation difficult. However, atrophic types of acne scars are by far the commonest [2].

Different treatment modalities have been under trial for treating them such as chemical peels, laser-assisted resurfacing, dermabrasion and microneedling (MN). However, none of them has proved optimal efficacy and many combinations are still under investigation [3].

Percutaneous collagen induction therapy, commonly known as microneedling, has proven to be a well-tolerated, minimally invasive office procedure with a minimal downtime, considerable suitability for different skin types with minimal side effects and hence high degree of patient satisfaction. This technique acts through creating micro-channels/wounds inducing a wound healing cascade followed by deposition of newly formed collagen and elastic fibers and clinically evident as more tightened skin [4].

Moreover, MN offers the advantage of transdermal drug delivery bypassing the barrier of stratum corneum. Such a method of drug delivery allows a high degree of drug bioavailability, a rapid onset of action due to the rich capillary bed of the superficial dermis and a rapid onset of drug action. Beside being of a good safety profile, MN is painless and does not require much expertize [5].

New combinations are being introduced for enhancing efficacy of MN. Platelet-rich plasma (PRP) is the most famous of which [6–10]. PRP is a biological product containing more than five times the baseline platelet-concentrate in addition to various growth and bioactive factors that promote angiogenesis and cell proliferation and modulate inflammation [10].

Botulinum toxin type-A, well known for its muscle paralyzing activity for cosmetic concerns, appears to have an inhibitory effect on fibroblasts and collagen remodeling activity [11, 12]. Its emerging off-label uses as intradermally injected micro-dosed Botulinum toxin-A for aesthetic concerns, e.g., improving skin texture is an interesting area of research [13]. We thought of enhancing topical bioavailability of Botulinum toxin-A using MN and getting added benefit of such a combination.

In this study, we compare the efficacy of combined MN and topical PRP versus combined MN and topical Botulinum toxin-A in treating atrophic acne scars in a split face study design.

## Patients and methods

Thirty patients with atrophic acne scars were recruited from the attending patients at the outpatient clinic. All patients were offered a total of four treatment sessions with a one-month interval in between. They were followed up for 3 months after the last session.

All included patients were subjected to thorough dermatological examination to assess their skin type, scar types and scar severity according to Goodman and Baron qualitative global acne scarring grading system and the quartile grading scale. Patients demonstrating any of the following were excluded from the study:

- Active acne lesion.
- Keloidal tendency.
- Pregnancy and lactation.
- Active skin infection, e.g., herpes labialis.
- Patients who underwent any cosmetic facial procedure, especially Botulinum toxin-A injection, during the last 6 months.
- Patients with bleeding disorders.

Patients were informed thoroughly about the techniques used and their expected outcomes to avoid any unrealistic expectations. In a split face manner, all patients were subjected to MN followed immediately by topical PRP at the right side versus MN followed immediately by topical Botulinum toxin-A on the left side. MN was done using an electric MN device (dermapen, Bomtech Electronics/Korea (34, Hyoryeong-ro 49-gil, Seocho-gu, Seoul, JX-120DR) equipped with a 36-needle head using a 1.5 mm needles length at speed 3–4 according to treated scar types.

The pre-procedure care consisted of applying a thick layer of a topical anesthetic cream (Lidocaine 2.5% &Prilocaine 2.5%, Pridocaine^®^) over the area to be treated for 30 min followed by degreasing and sterilization using alcohol before starting the session. Digital color photographs of each patient’s left and right profiles were obtained before each session for comparison and results documentation.

Botulinum toxin-A vials were obtained from Refinex (QMed BioTech Corp, KC Pharmaceuticals). The 100U vial was diluted using 10 ml saline to reach a final concentration of 10U/ml. For PRP, 10 ml of autologous whole blood was collected into tubes containing acid citrate dextrose (ACD) and centrifuged at 1500 rpm for 10 min to get PRP at the top of the test tube. Then, the PRP was further centrifuged at 3700 rpm for 10 min at room temperature of 22 °C to obtain a platelet count 4.5times higher than the base line. Calcium gluconate was added as an activator (1:9), i.e., 1 ml of calcium gluconate in 9 ml of PRP.

### Procedure

With the patient placed in a supine position, head stable, using a uniform firm pressure throughout the procedure, needling was carried out in three passes in vertical, horizontal and both diagonal directions on each half of the face. Pinpoint bleeding was taken as the endpoint. Right after bleeding stops and face is cleaned using a saline soaked gauze, 1 ml of reconstituted Botulinum toxin-A (total of 10U) was applied to the left side of the face using a 30G insulin syringe and left unwashed for half an hour. 1 m of prepared PRP was applied to the right side of the face and left unwashed for half an hour.

The treating physician remained the same through all sessions and a new device head was used for each session. Post-procedure care consisted of instruction the patient to apply topical antibiotic and to wear sunscreen. Patients were followed up on the 3rd and 7th day after the session for any adverse effects.

Evaluation was done using:

- Pre-sessions and post-session assessment using qualitative global scarring grading system [14] and the quartile grading scale where two blinded dermatologists evaluated the photographs taken before treatment and after completion of the treatment (one month after the last session). Physicians assessed the results using quartile grading scale which categorizes the improvement as follows: excellent improvement greater than 75%; very good improvement of 50–74%; good improvement of 25–49% and mild or poor improvement less than 25%.
- Degree of pain: pain during the session was measured by VASI scale. Pain severity measured on a scale ranged from (0: no pain to 10), patients were asked to estimate the degree of pain during sessions as mild (2–4), moderate (4–8) and severe (8–10) pain.
- Patient satisfaction: a questionnaire was given to patients at the end of treatment to assess their degree of improvement as poor, good, very good and excellent.

### Statistical analysis

Data collected throughout history, basic clinical examination, laboratory investigations and outcome measures coded, entered and analyzed using Microsoft Excel software. Data were then imported into Statistical Package for the Social Sciences (SPSS version 20.0) (Statistical Package for the Social Sciences) software for analysis. According to the type of data qualitative represent as number and percentage, quantitative continues group represent by mean ± SD, the following tests were used to test differences for significance; difference and association of qualitative variable by Chi-square test (X2). *P* value was set at < 0.05 for significant results & < 0.001 for high significant result.

## Results

All included 30 patients (24 females and 6 males) completed their sessions with no dropouts. Their mean age was 26.7 ± 5.02. Regarding their skin photo-type, about half of the studied group (53.35%) had skin photo-type III while 30% of them had type IV. 13.3% had type II and only 3.3% were type I. Disease duration ranged from 2–13 months with a mean duration of 6.6 ± 2.7 months. 90.0% of them never had any treatment for their scars and none of them had keloids or history of them.

There was no statistically significant difference between the two sides regarding scar type with matched numbers of each scar type in both groups (43.3%, 23.3% & 56.7%) of the right (MN + PRP) and left (MN + Botulinum toxin-A) sides were boxcar, rolling and icepick, respectively.

**Fig. 1:**
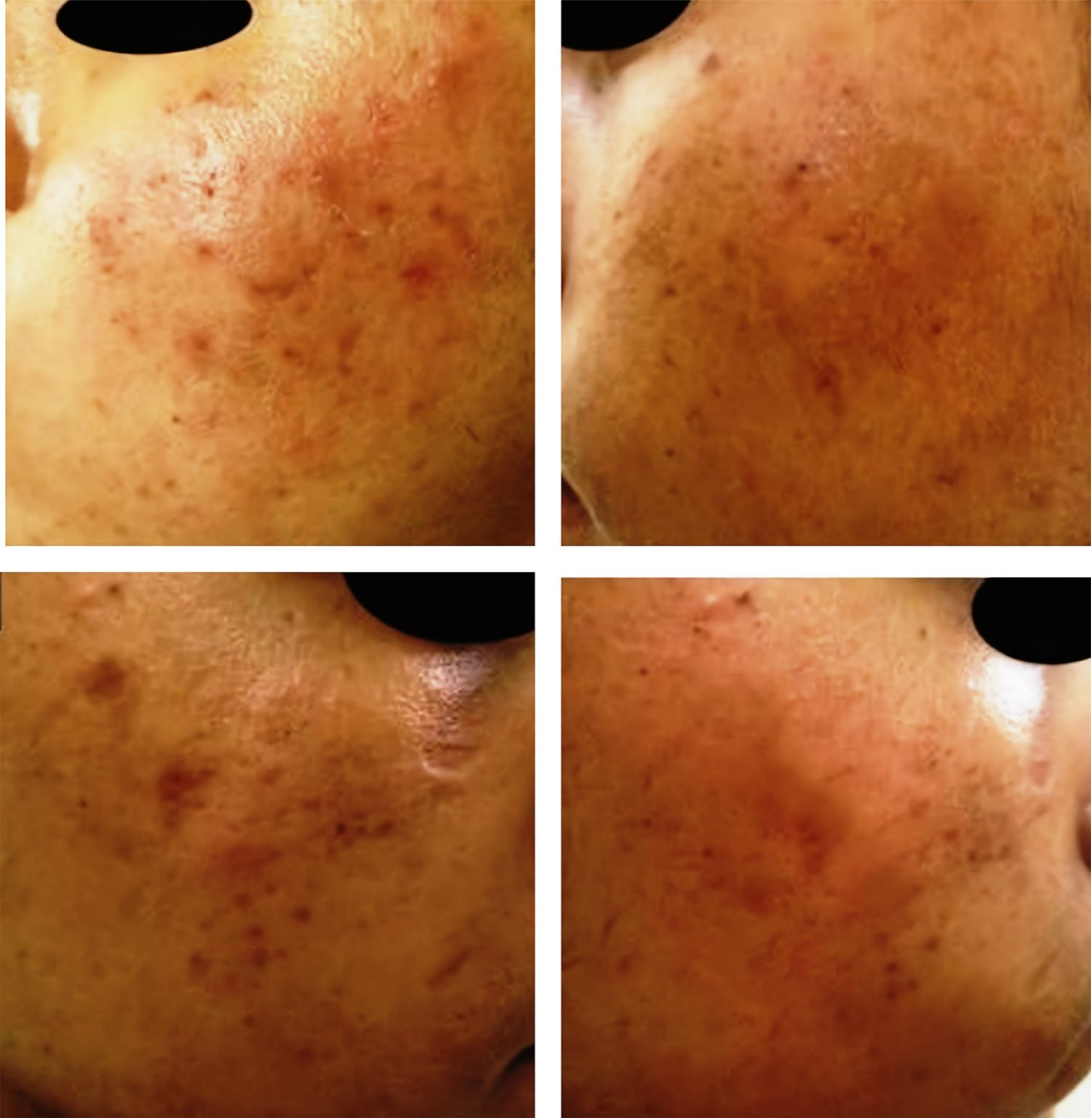
A 22-year-old male patient with rolling post-acne scars. Upper images showing excellent response of the scars on the left side of the face treated with MN + Botulinum toxin-A. Lower images showing excellent response of the scars on the right side of the face treated with MN + PRP

**Fig. 2:**
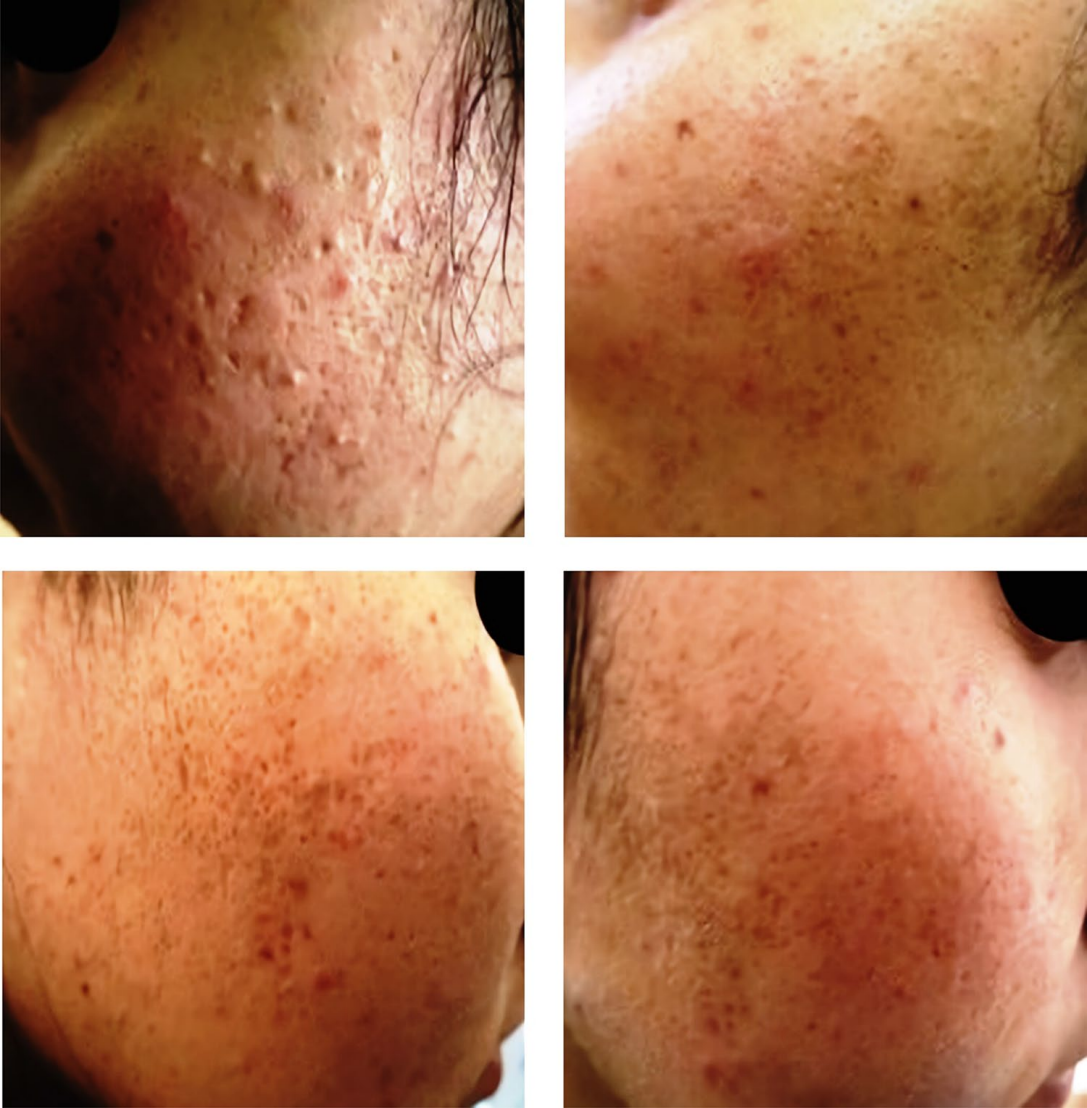
A 26-year-old female patient with ice-pick post-acne scars. Upper images showing a very good response of the scars on the left side of the face treated with MN + Botulinum toxin-A. Lower images showing a good response of the scars on the right side of the face treated with MN + PRP

**Fig. 3:**
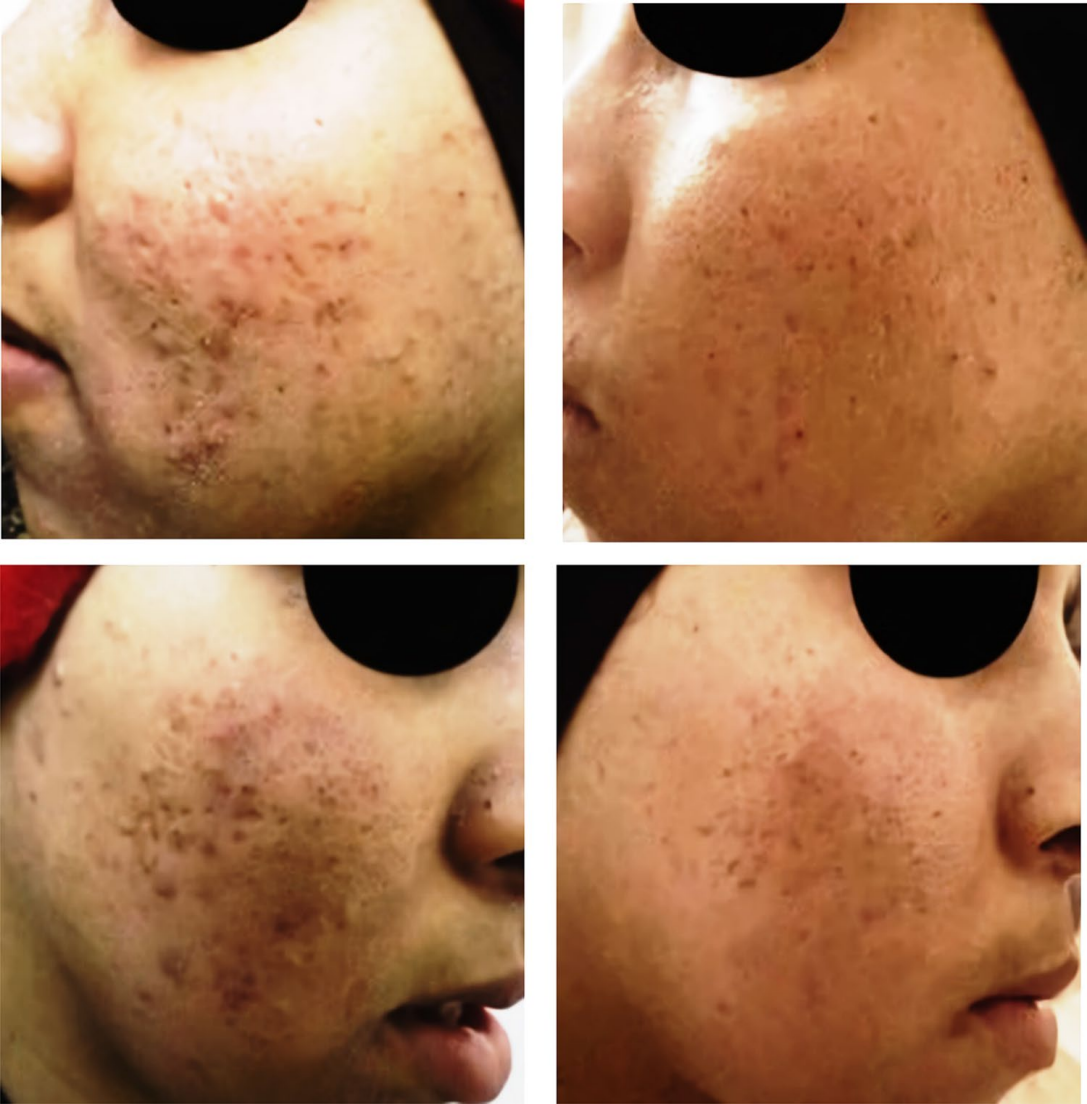
19-year-old male patient with boxcar post-acne scars. Upper images showing a good response of the scars on the left side of the face treated with MN + Botulinum toxin-A. Lower images showing a good response of the scars on the right side of the face treated with MN + PRP

Regarding response to therapy and according to the quartile grading scale, Table 1 shows there was no statistically significant difference between the two sides regarding degree of improvement where (23.4% & 13.3%) of the right and left sided, respectively, had an excellent response (Fig. 1). (30.0% & 36.7%) had a very good response (Fig. 2) while (33.3% & 30.0%) had a good response (Figs. 2, 3).

**Table 1:** Therapeutic response according to quartile grading scale

Improvement	Right side		Left side		*χ*^2^	*p*-value
	No (30)	%	No (30)	%		
Poor	4	13.3	6	20.0	1.5	0.6
Good	10	33.3	9	30.0		
Very good	9	30.0	11	36.7		
Excellent	4	23.4	4	13.3		

Chi-square test (*χ*^2^), Significant *P* value < 0.05

Regarding the difference in the qualitative global scarring grading system before and after treatment, there was a highly statistically significant improvement on both sides. Table 2 shows the two sides had the same qualitative global scarring grading system before treatment where nearly half of both sides (56.7%) of the right and left sided, respectively, were of Grade IV, 30.0% were grade III. Whilst after treatment, 30.0% & 26.7% were of grade II, 26.7% & 20.0 showed grade I and only 13.3% of both sides had grade IV.

**Table 2:** Therapeutic response according to qualitative global scaring grading system

The qualitative global scarring grading system	Right side		Left side		*χ*^2^	*p*-value
	No (30)	%	No (30)	%		
Before						
Grade I	0.0	0.0	0.0	0.0	–	–
Grade II	4	13.3	4	13.3		
Grade III	9	30.0	9	30.0		
Grade IV	17	56.7	17	56.7		
After						
Grade I	8	26.7	6	20.0		
Grade II	9	30.0	8	26.7	2.1	0.7
Grade III	9	30.0	12	40.0		
Grade IV	4	13.3	4	13.3		
*p*-value	0.001**		0.001**			

Chi-square test (*χ*^2^), *Significant *P*-value < 0.05, **Highly significant *P*-value < 0.001

According to Table 3, there was no statistically significant difference between the two sides regarding patient satisfaction where 36.7% & 46.7% of the right and left sides, respectively, had very good satisfaction and 36.7% & 30.0% had excellent satisfaction.

**Table 3:** Patient satisfaction after therapy

Patient satisfaction	Right side			Left side			*χ*^2^	*p*-value
	No (30)		%	No (30)		%		
Poor	1	3.3		1	3.3		0.6	0.9
Good	7	23.3		6	20.0			
Very good	11	36.7		14	46.7			
Excellent	11	36.7		9	30.0			

Chi-square test (*χ*^2^), *Significant *P*-value < 0.05, **Highly significant *P*-value < 0.001

There was no statistically significant difference relation between patients' age, sex, scar duration and skin photo-type with the degree of improvement according to quartile grading scale on both sides. But regarding the scar type, there was a statistically significant difference in the degree of improvement with different scar types where all of the excellent response (100.0%) were among the rolling scars while (83.3% and 44.4%) of the poor and good responses were among the boxcar type in the left sided group and most of the excellent response (85.7%) were among the rolling scars while (75.0% and 50.0%) of the poor and good responses were among the boxcar type in the right sided group.

## Discussion

Post-acne scars still constitutes a challenging problem facing dermatologists with no single standardized therapeutic modality. Their prevalence, high incidence of occurrence following moderate-to-severe acne and strong negative impact on patients’ quality of life are distressing for both patients and treating physicians. This study was conducted to evaluate the efficacy of two different combinations added to MN in treating post-acne scars. To the best of our knowledge, this is the first study to evaluate using Botulinum toxin-A in treatment of acne scars.

Skin needling therapy is a well-tolerated, minimally invasive office procedure for treatment of scars among other indications. MN creates dermal microchannels and micro-wounds that stimulate a wound healing cascade through the release of different growth promoting factors ending in neo-collagenesis and neovascularization. Moreover, those microchannels allow an easy clear way for topical agents to be more effectively absorbed [15].

Platelet-rich plasma is an autologous platelet concentrate in a small plasma volume that is rich in growth factors degranulated from its α-granules, e.g., vascular endothelial growth factor, platelet derived growth factor, epidermal growth factor and others that promote wound healing and remodeling. Its combined use with non-energy and non-invasive procedures has recently gained much attention in treating post-acne scars [16].

The first trial establishing the efficacy of combined MN and PRP was that by Fabbrocini and his colleagues [17] where they compared, in a split face fashion in only two sessions, the efficacy of MN alone (using dermaroller) versus MN combined with topical PRP. They showed great reduction of scar severity on both sides but with more prominent improvement on the side treated with combination therapy.

In 2014, Chawla and colleagues [6] showed superiority of combined MN and PRP over combined MN and topical vitamin-C where 37% of patients in vitamin C-treated group showed poor response in comparison to only 22% in the PRP-treated group. PRP has also been proven to be more effective in treating rolling and boxcar scars rather than icepick scars. Their results agree with our results regarding a slight better efficacy of PRP and the better response of rolling scar type.

Nofal et al. [18] showed combined PRP and MN, using dermaroller, in treating post-acne scars was of comparable efficacy and safety profile to intradermally injected PRP and CROSS technique. Ibrahim et al. (2018) found a significant improvement of acne scars for their two groups treated with MN alone and combined MN and PRP with insignificant difference between both groups.

Asif and his colleagues [7] did a comparative split face study in which they used combined MN and PRP on the right side versus combined MN and distilled water on the left side and found an excellent response in 40% and good response in 60% in the right sided group versus 10% and 6%, respectively, in the left sided group. They concluded that combining PRP to MN is significantly more efficacious than MN alone.

Amer et al. [9] found insignificant difference in scar improvement after treatment using combined MN and PRP versus combined MN and non-cross linked hyaluronic acid in a split face comparative study. Likewise, our results showed comparable efficacy of PRP and Botulinum toxin-A when combined with MN.

Platelet-rich plasma has also shown efficacy as an adjuvant to fractional Co2 Laser (FCL). Abdel-Maguid and colleagues [19] compared the effectiveness of combined FCL and PRP versus combined FCL and topical stem cell-conditioned media (SC-CM). They ended up showing no significant difference between both sides but with better and faster improvement on the PRP-treated side, like our results. On a molecular level, dermal collagen and procollagen type-1 gene were upregulated on both sides in comparison with FCL alone-treated sides.

Botulinum toxin-A is a neurotoxin obtained from the anerobic Clostridium Botulinum. Its use in the field in dermatology has expanded beyond its muscle paralyzing effect improving kinetic wrinkles giving a strong rejuvenating effect. It has shown a scar modulating effect in treating surgical and keloidal scars [20–24].

Off-label uses of Botulinum toxin-A is still a wide area for research. One of which is the “micro-dosing” technique which entails a sub- or intradermal injection of a lower concentration of Botulinum toxin-A than that used for the traditional intramuscular injection [25]. Such a technique and dosage are sufficient to cause weakening of superficial muscle fibers with dermal attachment thus releasing any pulling effect exerted on acne scars and that might explain the excellent response encountered with rolling scars.

This mechanism of action enhances the ability of MN to break fibrous strands stimulating a cascade of new collagen synthesis. We believed MN is more suitable to be combined with Botulinum toxin-A for treating acne scars being less painful than intradermal injection that requires expert skills to avoid any deeper injection resulting in muscle paralysis.

Sang OH [26] studied the in vitro effects of Botulinum toxin-A on normal fibroblasts and found that Botulinum toxin-A has a significant effect in increasing the level of collagen production and downregulating its degradation. Moreover, Botulinum toxin-A was found to enhance angiogenesis, attenuate fibrosis without affecting the epithelialization cascade of wound healing [27, 28]

In a randomized controlled trial for patients with periorbital wrinkles, treatment was performed with FCL followed by topical Botulinum toxin-A on one side and isotonic saline as control on the other side. There was a clinically significant greater degree of improvement in wrinkles on the Botulinum toxin-A treated side proving the concept that stratum corneum disruption with a fractional CO2 laser permits penetration of topical Botulinum toxin-A into the superficial fibers of orbicularis oculi [29].

In the same manner, MN was employed in our study to allow maximum Botulinum toxin-A penetration and precise dermal delivery to the treated site through disruption of the stratum corneum. MN, compared to fractional laser, is less costly, requires less training with less side effects and minimal downtime [30].

Regarding adverse effects, no major side effects were noticed in both groups except for a tolerable degree of pain and a short downtime. Both procedures were used safely with no post-procedure pigmentary problems in dark-skinned subjects. No side effect related to the muscle paralyzing action of Botulinum toxin-A were encountered as we used a lower concentration of Botulinum toxin-A than that used for treating wrinkles and a safer topical transdermal delivery vehicle through the microchannels created by MN thus only reaching the superficial muscle fibers attached to the dermis.

In conclusion, we present in this study a new proof of concept that combined therapy provide an efficient well-tolerated modality combining the advantages of different therapies acting synergistically with fewer side effects. The two modalities used inhere show comparable efficacy with a wide safety profile for an effective office procedure. An advantage may go to PRP being of lower cost. A larger number of patients may be needed for further confirmation of results and meaningful statistical analysis.

## Data Availability

All data generated and analyzed during this study are included in the published article and any supplementary material.

## Abbreviations

MN: Microneedling
PRP: Platelet-rich plasma
