# Correction to: Microneedling combined with botulinum toxin‑A versus microneedling combined with platelet‑rich plasma in treatment of atrophic acne scars: a comparative split face study

**DOI:** 10.1007/s00403-023-02731-1

**Published:** 2023-10-21

**Authors:** Waleed Albalat, Soheir Ghonemy, Ayat Saleh, Mona Elradi

**Affiliations:** Dermatology, Venereology & Andrology Department, Zagazig University Hospitals, Zagazig University, Zagazig, Egypt

**Correction to: Archives of Dermatological Research (2023) 315:839–846** 10.1007/s00403-022-02446-9

In this article the Refinex’s product, botulinum toxin was incorrectly mentioned as “Botox” but should have been “Botulinum toxin-A”. The corrected sentences are provided below.

In the methods section under “Abstract”, the sentence beginning “Right side of the face..” should have read “Right side of the face was treated with microneedling and platelet-rich plasma while the left side was treated microneedling and Botulinum toxin-A.”

The Keywords should have read as Acne · Scars · Microneedling · PRP · Botulinum toxin-A

The eighth and ninth paragraph under “Introduction” should have read as follows:

Botulinum toxin type-A, well known for its muscle paralyzing activity for cosmetic concerns, appears to have an inhibitory effect on fibroblasts and collagen remodeling activity [11, 12]. Its emerging off-label uses as intradermally injected micro-dosed Botulinum toxin-A for aesthetic concerns, e.g., improving skin texture is an interesting area of research [13]. We thought of enhancing topical bioavailability of Botulinum toxin-A using MN and getting added benefit of such a combination.

In this study, we compare the efficacy of combined MN and topical PRP versus combined MN and topical Botulinum toxin-A in treating atrophic acne scars in a split face study design.

The fifth point in the list under “Patients and methods” should have read “Patients who underwent any cosmetic facial procedure, especially Botulinum toxin-A injection, during the last 6 months.”

The sentence beginning “In a split face manner,...” should have read “In a split face manner, all patients were subjected to MN followed immediately by topical PRP at the right side versus MN followed immediately by topical Botulinum toxin-A on the left side.”

The sentence beginning “Botox vials were obtained from Refinex…” should have read “Botulinum toxin-A vials were obtained from Refinex (QMed BioTech Corp, KC Pharmaceuticals).”

The sentence beginning “Right after bleeding stops and face…” should have read “Right after bleeding stops and face is cleaned using a saline soaked gauze, 1 ml of reconstituted Botulinum toxin-A (total of 10U) was applied to the left side of the face using a 30G insulin syringe and left unwashed for half an hour.”

The second paragraph under “Results” should have read as follows:

There was no statistically significant difference between the two sides regarding scar type with matched numbers of each scar type in both groups (43.3%, 23.3% & 56.7%) of the right (MN + PRP) and left (MN + Botulinum toxin-A) sides were boxcar, rolling and icepick, respectively.

Figure 1 caption should have read as “A 22-year-old male patient with rolling post-acne scars. Upper images showing excellent response of the scars on the left side of the face treated with MN + Botulinum toxin-A. Lower images showing excellent response of the scars on the right side of the face treated with MN + PRP.”

The last sentence in the first paragraph under “Discussion” should have read as “To the best of our knowledge, this is the first study to evaluate using Botulinum toxin-A in treatment of acne scars.”

Figure 2 caption should have read as “A 26-year-old female patient with ice-pick post-acne scars. Upper images showing a very good response of the scars on the left side of the face treated with MN + Botulinum toxin-A. Lower images showing a good response of the scars on the right side of the face treated with MN + PRP.”

The last sentence in the eighth paragraph under “Discussion” should have read as “Likewise, our results showed comparable efficacy of PRP and Botulinum toxin-A when combined with MN.”

Figure 3 caption should have read as “19-year-old male patient with boxcar post-acne scars. Upper images showing a good response of the scars on the left side of the face treated with MN + Botulinum toxin-A. Lower images showing a good response of the scars on the right side of the face treated with MN + PRP.”

The first sentence in the tenth paragraph under “Discussion” should have read as “Botulinum toxin-A is a neurotoxin obtained from the anerobic Clostridium Botulinum.”

The last few paragraphs under “Discussion” should have read as follows:

Off-label uses of Botulinum toxin-A is still a wide area for research. One of which is the “micro-dosing” technique which entails a sub- or intradermal injection of a lower concentration of Botulinum toxin-A than that used for the traditional intramuscular injection [25]. Such a technique and dosage are sufficient to cause weakening of superficial muscle fibers with dermal attachment thus releasing any pulling effect exerted on acne scars and that might explain the excellent response encountered with rolling scars.

This mechanism of action enhances the ability of MN to break fibrous strands stimulating a cascade of new collagen synthesis. We believed MN is more suitable to be combined with Botulinum toxin-A for treating acne scars being less painful than intradermal injection that requires expert skills to avoid any deeper injection resulting in muscle paralysis.

Sang OH [26] studied the in vitro effects of Botulinum toxin-A on normal fibroblasts and found that Botulinum toxin-A has a significant effect in increasing the level of collagen production and down- regulating its degradation. Moreover, Botulinum toxin-A was found to enhance angiogenesis, attenuate fibrosis without affecting the epithelialization cascade of wound healing [27, 28].

In a randomized controlled trial for patients with periorbital wrinkles, treatment was performed with FCL followed by topical Botulinum toxin-A on one side and isotonic saline as control on the other side. There was a clinically significant greater degree of improvement in wrinkles on the Botulinum toxin-A treated sideproving the concept that stratum corneum disruption with a fractional CO2 laser permits penetration of topical Botulinum toxin-A into the superficial fibers of orbicularis oculi [29].

In the same manner, MN was employed in our study to allow maximum Botulinum toxin-A penetration and precise dermal delivery to the treated site through disruption of the stratum corneum. MN, compared to fractional laser, is less costly, requires less training with less side effects and minimal downtime [30].

Regarding adverse effects, no major side effects were noticed in both groups except for a tolerable degree of pain and a short downtime. Both procedures were used safely with no post-procedure pigmentary problems in dark skinned subjects. No side effect related to the muscle paralyzing action of Botulinum toxin-A were encountered as we used a lower concentration of Botulinum toxin-A than that used for treating wrinkles and a safer topical transdermal delivery vehicle through the microchannels created by MN thus only reaching the superficial muscle fibers attached to the dermis.

The original article has been corrected.

